# The interaction of canonical Wnt/β-catenin signaling with protein lysine acetylation

**DOI:** 10.1186/s11658-021-00305-5

**Published:** 2022-01-15

**Authors:** Hongjuan You, Qi Li, Delong Kong, Xiangye Liu, Fanyun Kong, Kuiyang Zheng, Renxian Tang

**Affiliations:** 1 Jiangsu Key Laboratory of Immunity and Metabolism, Department of Pathogenic Biology and Immunology, Xuzhou Medical University, Xuzhou, Jiangsu China; 2 Laboratory Department, The People’s Hospital of Funing, Yancheng, Jiangsu China; 3 National Demonstration Center for Experimental Basic Medical Sciences Education, Xuzhou Medical University, Xuzhou, Jiangsu China

**Keywords:** Protein lysine acetylation, Canonical Wnt/β-catenin signaling, Interaction, Therapy, Molecular mechanisms

## Abstract

Canonical Wnt/β-catenin signaling is a complex cell-communication mechanism that has a central role in the progression of various cancers. The cellular factors that participate in the regulation of this signaling are still not fully elucidated. Lysine acetylation is a significant protein modification which facilitates reversible regulation of the target protein function dependent on the activity of lysine acetyltransferases (KATs) and the catalytic function of lysine deacetylases (KDACs). Protein lysine acetylation has been classified into histone acetylation and non-histone protein acetylation. Histone acetylation is a kind of epigenetic modification, and it can modulate the transcription of important biological molecules in Wnt/β-catenin signaling. Additionally, as a type of post-translational modification, non-histone acetylation directly alters the function of the core molecules in Wnt/β-catenin signaling. Conversely, this signaling can regulate the expression and function of target molecules based on histone or non-histone protein acetylation. To date, various inhibitors targeting KATs and KDACs have been discovered, and some of these inhibitors exert their anti-tumor activity via blocking Wnt/β-catenin signaling. Here, we discuss the available evidence in understanding the complicated interaction of protein lysine acetylation with Wnt/β-catenin signaling, and lysine acetylation as a new target for cancer therapy via controlling this signaling.

## Introduction

Canonical Wnt/β-catenin signaling is one of the well-known conserved cell-communication mechanisms that involve the growth, metastasis, stemness maintenance, and therapeutic resistance in different kinds of cancer [1, 2]. Especially, β-catenin is one core molecule of this signaling. With the absence of extracellular Wnt signals (Wnt off state), β-catenin is restricted by a “destruction protein complex”, which consists of casein kinase 1 (CK1), glycogen synthase kinase 3β (GSK3β), Axin, and adenomatous polyposis coli (APC) molecules, and sequentially degraded by the E3 ubiquitin ligase subunit beta-transducin repeat-containing protein (β-TRCP) through ubiquitination in the cytoplasm. However, after the secreted Wnt molecules bind to Frizzled proteins (FZD) and lipoprotein receptor-related protein (LRP) 5/6 receptor complex (Wnt on state), the activated signals recruit Dishevelled (DVL) and Axin to the FZD-LRP5/6 co-receptors to disrupt the “destruction protein complex”. Subsequently, β-catenin is released from the complex and translocates into the cell nucleus, where β-catenin forms a complex with T cell factor (TCF)/lymphoid enhancer factor (LEF) to activate the expression of Wnt-dependent genes, including MYC as well as cyclin D1 (CCND1) genes. Also, this signaling can be inhibited by endogenous inhibitory molecules, including Wnt inhibitory factor 1 (WIF-1), Dickkopf-related protein (DKK), and secreted frizzled-related proteins (SFRPs). Particularly, WIF-1 and SFRPs directly interact with Wnts, and DKK blocks the FZD-LRP5/6 receptor complex to inhibit Wnt/β-catenin signaling [3, 4]. Furthermore, numerous cellular factors, including protein kinases [5], non-coding RNA [6], and different posttranslational modifications (PTM) [7], including phosphorylation, sumoylation, and ubiquitination, are identified to play a vital role in modulating this signaling.

As an evolutionarily conserved protein modification, lysine acetylation can transfer the acetyl group from acetyl-coenzyme A to target substrates to alter their biological functions [8]. Until now, the acetylation of histone, as well as non-histone proteins, has been discovered [9]. Also, the acetylation levels in most identified histone and non-histone proteins rely on lysine acetyltransferases (KATs, also named histone acetyltransferases, HATs), and lysine deacetylases (KDACs, also called histone deacetylases, HDACs). KATs are further divided into cytoplasmic and nuclear KATs. Recently, tubulin N-acetyltransferase 1 (TAT1) and KAT1 have been discovered to act as cytoplasmic KATs. Nuclear KATs are classified into 5 families: CREB-binding protein (CBP)/p300, basal transcription factors, MYST, general control non-repressed 5 (GCN5)/CBP-associated factor (PCAF), and nuclear receptor coactivator family. In addition, KDACs are divided into class I (HDAC1, 2, 3, 8) [8, 9], class II (HDAC4, 5, 6, 9, 10) [10, 12], class III (sirtuin (SIRT)1, 2, 3, 4, 5, 6, 7) [11], and class IV (HDAC11) [12]. Especially, it is proved that histone acetylation facilitates the transcription of target genes [8]. Non-histone protein acetylation is responsible for the modulation of various molecular functions, including protein stability and enzymatic activity [12].

Increasing evidence indicates that the protein lysine acetylation, including the acetylation of histones as well as non-histone proteins, is vital for Wnt/β-catenin signaling activation. Conversely, this signaling can regulate the protein lysine acetylation. Here, we discuss the latest advances related to protein lysine acetylation to regulate Wnt/β-catenin signaling, the effect of this signaling on controlling protein lysine acetylation, as well as the potential of targeting lysine acetylation to inhibit this signaling to facilitate cancer treatment.

### The function of Wnt/β-catenin signaling in non-histone protein acetylation

As mentioned above, the effect of Wnt/β-catenin signaling in different biological processes is mainly dependent on Wnt target genes [1]. However, current evidence indicates that this signaling also controls multiple molecular functions by modulating the acetylation levels of target proteins. For example, p53 plays a fundamental role in various biological processes, but the mechanisms associated with the regulation of p53 are still not fully understood. The study by Riascos-Bernal et al. showed that β-catenin can suppress the function of p53 via inhibiting its acetylation mediated by CBP [13]. NF-κB signaling is a vital regulator of inflammation. Especially, the acetylation of RelA, a core molecule in NF-κB signaling, could be acetylated by CBP. However, β-catenin can inhibit RelA acetylation to restrict NF-κB target gene expression in lung fibroblast and carcinoma cells to further inhibit inflammation [14].

In Wnt/β-catenin signaling, as a restriction factor, GSK3β also could regulate the acetylation levels of different molecules. Eom et al. reported that GSK3β can bind to p53, and the interaction is capable of initiating K373 and K382 acetylation in p53 [15]. However, it is still unknown which among the KATs contributes to the increase of p53 acetylation induced by GSK3β. In addition to p53, GSK3 also could interact with and phosphorylate 60 kDa Tat-interactive protein (TIP60), and then strengthen Unc51-like kinase-1 (ULK1) acetylation mediated by TIP60 to facilitate autophagy [16]. However, whether other molecules in this signaling participate in the modulation of non-histone acetylation is largely unknown.

### The contribution of non-histone protein acetylation to Wnt/β-catenin signaling

**Fig. 1:**
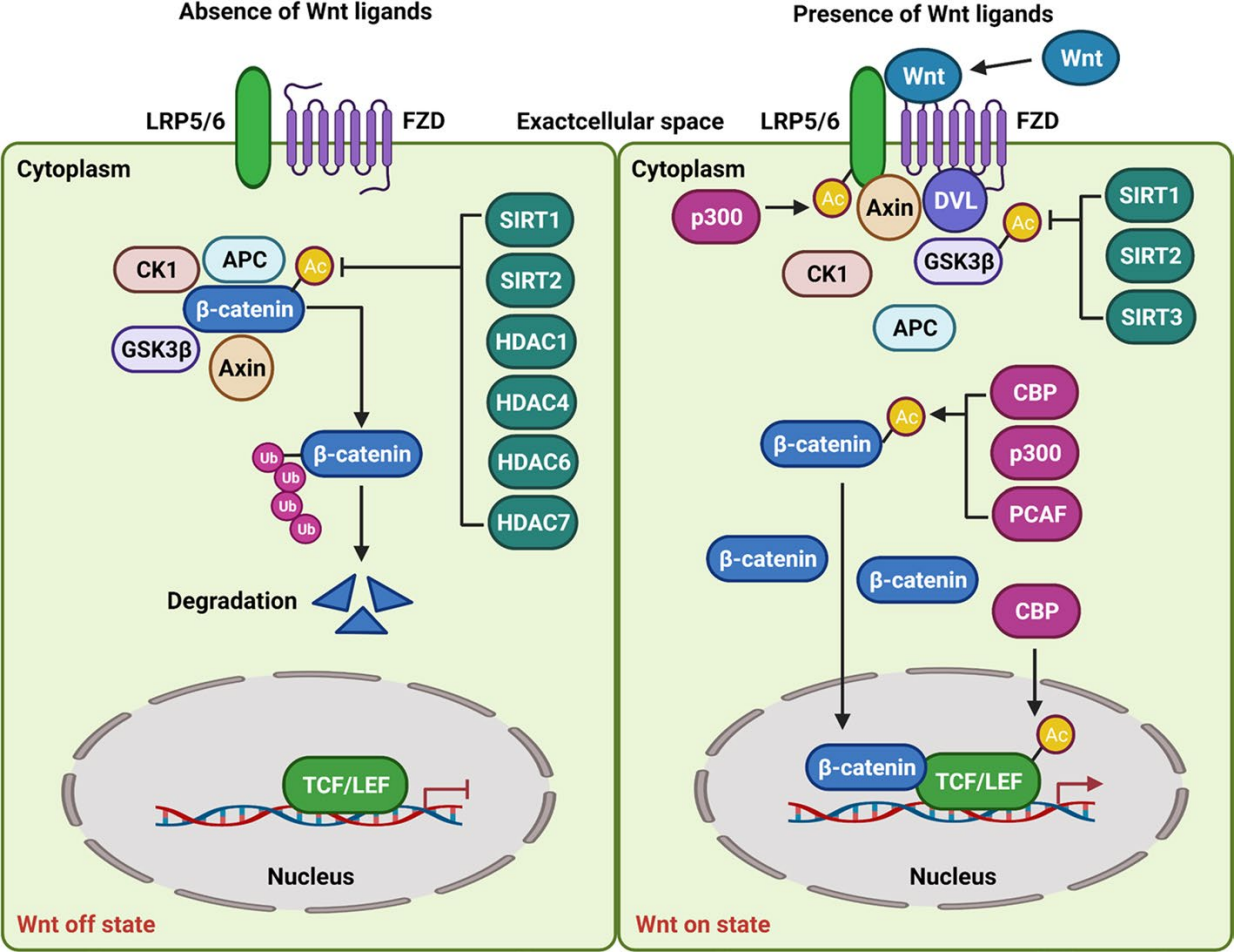
Regulation of non-histone acetylation on canonical Wnt/β-catenin signaling. Without the binding of Wnt molecules to the FZD-LRP5/6 co-receptor complex, the signaling is inactive (Wnt off state). During the Wnt off state, the destruction complex composed of GSK3β, APC, Axin1, and CK1, can interact with β-catenin, leading to its degradation with ubiquitin–proteasome in the cytoplasm. When Wnt molecules interact with the FZD-LRP5/6 co-receptor (Wnt on state), the complex recruits Axin and DVL to induce the release of β-catenin from the destruction complex and enhance its accumulation as well as nuclear translocation. In the cell nucleus, β-catenin interacts with LEF/TCF and further activates Wnt-dependent gene transcription. During the Wnt off state, acetylation of β-catenin can be inhibited by SIRT1, SIRT2, HDAC1, HDAC2, HDAC4, HDAC6, and HDAC67 to block its activity. During the Wnt on state, LRP6 is acetylated by p300 to facilitate signaling activation. Then, β-catenin is capable of being acetylated by CBP, p300, and PCAF to increase protein activity. Also, acetylation of GSK3β is suppressed by SIRT1, SIRT2, and SIRT3. TCF is acetylated by CBP

So far, in Wnt/β-catenin signaling, the acetylation of four molecules, including LRP6, TCF4, GSK3β, and β-catenin has been unveiled (Fig. 1). For example, Wu et al. reported that p300 is capable of facilitating LRP6 acetylation and then triggering its phosphorylation to sensitize this signaling and further accelerate the self-renewal of colorectal cancer cells to facilitate liver metastasis [17]. As for TCF4, it has been shown that, based on CBP, the protein can be acetylated at K150. Furthermore, the conformational change of the TCF4-DNA complex can be induced by acetylated TCF4 [18]. As one component of the “destruction protein complex” in this signaling, GSK3β is also reported to be acetylated, and the results show that SIRT1, SIRT2, and SIRT3 could inhibit GSK3β to block its activity [19–21]. However, whether other molecules in the “destruction protein complex”, including CK1, Axin, and APC, are capable of being modulated by acetylation is still unclear.

To date, β-catenin acetylation has been widely explored by different groups. It has been found that β-catenin acetylation is relevant to CBP, p300, and PCAF. Especially, the acetylation of K345 in β-catenin is associated with p300 [22]. The K49 in β-catenin can be acetylated by CBP [23]. The K19 and K49 in β-catenin are the critical residues for PCAF-mediated acetylation [24]. Furthermore, β-catenin acetylation not only improves its stability by inhibiting the ubiquitin-mediated degradation [25] but also induces its nuclear translocation, to enhance its binding to TCF and further enhance the transcription of Wnt-dependent genes [22].

Moreover, dependent on HATs as mentioned above, multiple molecules are involved in modulating β-catenin acetylation. For instance, Li et al. found that blocking proliferation 1 (BOP1) can initiate β-catenin acetylation that is dependent on CBP to strengthen the drug resistance of breast cancer [26]. Forkhead box protein P1 (FOXP1) has been proven to activate this signaling by increasing β-catenin acetylation in different biological processes [27, 28]. Especially, in B cell lymphoma, FOXP1 can enhance β-catenin acetylation through CBP. Next, the increased acetylation of β-catenin benefits the gene transcription mediated by FOXP1 [28]. In addition to BOP1 and FOXP1, Zhang et al. found that cell-cycle related and expression-elevated protein in tumor (CREPT) facilitates colorectal cancer growth by enhancing p300-mediated β-catenin acetylation [29]. In addition, high glucose-dependent β-catenin nuclear retention also requires p300-dependent β-catenin acetylation at K354 to trigger the increase of MYC as well as CCND1 genes in multiple cancers [30]. Also, ATP citrate lyase (ACLY) is found to affect β-catenin acetylation at K49 in hepatoma carcinoma (HCC) cells [31]. Additionally, Wnt1 and Wnt7b [32] also can accelerate β-catenin acetylation at K49. However, it is still unknown which of the HATs contribute to β-catenin acetylation mediated by Wnt molecules.

Although several molecules mentioned above contribute to β-catenin acetylation, current reports show that other cellular factors can inhibit the acetylation of β-catenin by suppressing KATs. For example, the transcription factor Kruppel-like factor 4 (KLF4) is critical for intestinal differentiation. Moreover, the differentiation mediated by KLF4 is observed to rely on its interaction with β-catenin to inhibit acetylation of the protein mediated by p300/CBP [33]. The nuclear factor of activated T-cells 5 (NFAT5) is also verified to take participate in repressing Wnt/β-catenin signaling. Especially, NFAT5 directly binds to β-catenin and inhibits the interaction of β-catenin with CBP to block its acetylation [34].

Apart from KATs, many molecules are capable of restricting β-catenin acetylation by KDACs, including SIRT1, SIRT2, HDAC1, HDAC4, HDAC6, and HDAC7. For example, in bladder cancer cells, Capsaicin can attenuate cell migration via SIRT1-dependent inhibition of β-catenin acetylation [35]. Additionally, it is observed that the expression of β-catenin is increased in liver cancer stem cells (LCSCs) and its expression is particularly associated with SIRT1. Moreover, the decrease of β-catenin acetylation mediated by SIRT1 can regulate the self-renewal of LCSCs [36]. Also, SIRT1 can deacetylate β-catenin to block the transcription of Wnt-dependent genes and regulate cell differentiation [37–39]. In addition to SIRT1, SIRT2 also could inhibit β-catenin acetylation to suppress the levels of MYC and CCND1 [40].

Recently, Chen et al. reported that B-cell lymphoma 3 (Bcl-3) can maintain K49 acetylation in β-catenin. Mechanistically, Bcl-3-dependent suppression of interaction between HDAC1 and β-catenin is associated with increased β-catenin acetylation in colorectal cancer [41]. Also, HDAC4 can inhibit β-catenin acetylation to increase its ubiquitination [42]. Interestingly, in breast cancer cells, it has been found that HDAC6 not only can inhibit K49 acetylation in β-catenin but also facilitate K345 acetylation suppression in β-catenin [43, 44]. Additionally, the interaction of HDAC7 with β-catenin also causes a decrease of K49 acetylation in β-catenin in glioma cells [45].

### The influence of Wnt/β-catenin signaling on histone acetylation

**Fig. 2:**
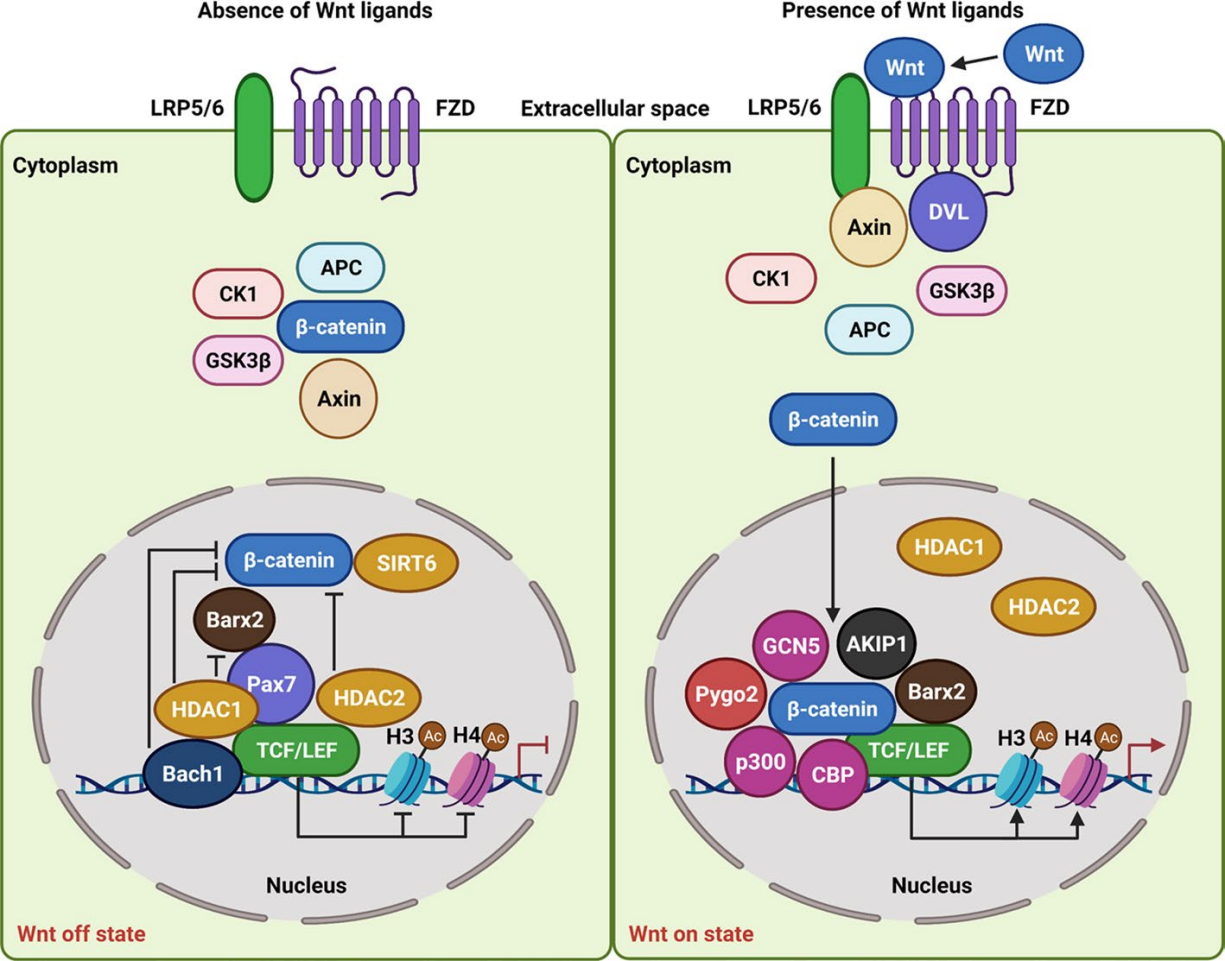
Molecular mechanisms associated with histone acetylation in β-catenin-TCF/LEF-dependent transcription of Wnt target genes. In the Wnt off state, TCF/LEF interacts with HADC1 and HADC2 to inhibit gene transcription by inhibiting acetylation of histone H3/H4. SIRT6 binds to β-catenin and participates in inhibition of β-catenin-TCF/LEF-dependent transcription. Bach1 binds to HDAC1 to suppress β-catenin. Pax7 inhibits the function of Barx2 in activating β-catenin, to block histone acetylation-associated gene transcription. In the Wnt on state, β-catenin interacts with TCF/LEF to release HDAC1 and HDAC2 to transcription factor binding sites. Additionally, β-catenin recruits p300, CBP, AKIP1, and Barx2 to facilitate histone acetylation at the target gene promoter. Pygo2 is also recruited by β-catenin to bind to p300 and GCN5 to promote histone acetylation to induce gene expression

As mentioned, after Wnt/β-catenin signaling sensitization, β-catenin can interact with and activate TCF/LEF, which is also controlled by various transcriptional coregulators, including the coactivators and corepressors, at the target gene promoter in the cell nucleus [46, 47]. Without Wnt signals, TCF/LEF binds to HDAC1 and HDAC2, and causes histone H3 hypo-acetylation at the promoters of Wnt-dependent genes, and induces suppression of gene transcription [48–51]. Moreover, HDACs are capable of inhibiting β-catenin by enhancing its degradation, and inhibiting its nuclear translocation to regulate downstream gene transcription (Fig. 2) [52]. Also, SIRT6, one component of HDACs, can bind to β-catenin, resulting in histone H3K56 deacetylation to prevent gene transcription [51]. In addition to HDAC1, HDAC2, and SIRT6, whether other constituents of HDACs take part in the transcriptional inhibition through regulating the function of β-catenin or TCF/LEF is not fully clarified.

Upon activation by Wnt molecules, β-catenin is capable of interacting with TCF/LEF and disrupting the interaction of TCF/LEF with HDACs to form an active transcriptional complex in the cell nucleus (Fig. 2). Meanwhile, β-catenin can recruit p300/CBP to the transcriptional complex. In turn, p300/CBP acts as the transcriptional coactivator of β-catenin to induce histone H4 acetylation and stimulate gene transcription [53, 54]. As a chromatin effector, Pygopus 2 (Pygo2) is capable of being acetylated by the protein complex composed of CBP and p300. In the nucleus, when Pygo2 binds to the β-catenin-TCF/LEF complex, the acetylation of Pygo2 facilitates histone H3/H4 acetylation by recruiting CBP/p300 and GCN5 to activate gene transcription [55]. In HCC cells, A-kinase interacting protein 1 (AKIP1) can bind to β-catenin and maintain its accumulation in the cell nucleus by suppressing its binding to APC. Moreover, AKIP1 has the capability of enhancing β-catenin activation and leading to the recruitment of CBP to activate gene transcription [56].

However, in the nucleus, BTB domain and CNC homolog 1 (Bach1) is found to directly bind to TCF4, and the interaction not only reduces the binding of β-catenin to TCF4 but also decreases the interaction of p300/CBP with β-catenin to inhibit β-catenin acetylation. Furthermore, Bach1 can occupy the TCF4-binding site and recruit HDAC1 to the target genes promoter [57]. BarH-like Homeobox 2 (Barx2) and paired box 7 (Pax7) are recently identified components of the TCF/LEF transcriptional complex. After Wnt3a stimulation, Barx2 can be recruited to TCF/LEF binding sites with glutamate receptor interacting protein 1 (GRIP-1) at the Axin2 promoter, and further recruit β-catenin to induce H3 acetylation in myoblasts. In contrast, dependent on the interaction with corepressor HDAC1, Pax7 is able to repress Axin2 promoter activity, through inhibiting H3 acetylation at the promoter of Axin2 mediated by Barx2 at the transcriptional complex [58]. In myoblasts, Wnt3a not only induces expression of Barx2 at the gene level but also stabilizes Barx2 at the protein level to facilitate Axin2 expression. Conversely, Wnt3a can suppress Pax7 protein expression to induce transcription of the Wnt target gene Axin2.

### The impact of histone acetylation on Wnt/β-catenin signaling

To date, increasingly studies have indicated that the activation of histone acetylation at Wnt, β-catenin, and FZD promoters can control their transcription (Table 1). In detail, the results from Jing et al. show that the levels of histone H3K9 acetylation at Wnt1, Wnt10a, Wnt6, and Wnt10b promoters are decreased in bone marrow-derived mesenchymal stem cells (BMSCs). Conversely, GCN5 is observed to be responsible for the differentiation of BMSCs by increasing H3K9 acetylation at the promoters of these Wnt genes to facilitate Wnt/β-catenin signaling activation [59]. As for β-catenin, Huang et al. found that, by interacting with the β-catenin promoter, HDAC1 can inhibit its expression in mouse embryonic fibroblasts (MEF, C3H10T1/2 cells). However, it is still unclear which histones at the promoter of β-catenin could be regulated by HDAC1 to suppress its gene expression [60]. In an Alzheimer’s disease mouse model, the nuclear paraspeckle assembly transcript 1 (NEAT1) can enhance FZD3 transcription by increasing the acetylation of H3K27 at its promoter. Mechanistically, based on NEAT1, P300 could be recruited to the FZD3 promoter and enhance the transcription of the FZD3 gene through histone H3K27 acetylation [61]. Additionally, Liu et al. found that SIRT6 can interact with FZD4 and suppresses FZD4 transcription by decreasing histone H3K9 acetylation in hepatoblastoma cells [62].

**Table 1:** Histone acetylation and modulation of molecules in Wnt/β-catenin signaling

Histone modifier	Histone modification	Target gene	Effect on target genes	Target cells or models	References
GCN5	H3K9Ac	Wnt1	Activation	BMSCs	[59]
GCN5	H3K9Ac	Wnt10a	Activation	BMSCs	[59]
GCN5	H3K9Ac	Wnt6	Activation	BMSCs	[59]
GCN5	H3K9Ac	Wnt10b	Activation	BMSCs	[59]
HDAC1	unknown	β-catenin	Inhibition	MEFs	[60]
P300	H3K27Ac	FZD3	Activation	Alzheimer’s disease mouse model	[61]
SIRT6	H3K9Ac	FZD4	Inhibition	Hepatoblastoma cells	[62]
HDAC3	unknown	WIF-1	Inhibition	Fibroblasts	[63]
HDAC1	H3Ac	SFRP1	Inhibition	HCC cells	[64]
SIRT1	H3K9Ac	SFRP1	Inhibition	MEFs	[38]
SIRT1	H4K16Ac	SFRP1	Inhibition	MEFs	[38]
SIRT1	H3K9Ac	SFRP2	Inhibition	MEFs	[38]
SIRT1	H4K16Ac	SFRP2	Inhibition	MEFs	[38]
HDAC1	H3Ac	DKK1	Inhibition	Breast cancer cells	[66]
HDAC2	H3Ac	DKK1	Inhibition	Breast cancer cells	[66]
HDAC1	H4Ac	DKK1	Inhibition	Breast cancer cells	[66]
HDAC2	H4Ac	DKK1	Inhibition	Breast cancer cells	[66]
GCN5	H3K9Ac	DKK1	Activation	Periodontal ligament stem cells	[67]
GCN5	H3K14Ac	DKK1	Activation	Periodontal ligament stem cells	[67]
p300	H3Ac	DKK1	Activation	Breast cancer cells, HCC cells,	[68, 70]
CBP	H3Ac	DKK1	Activation	Breast cancer cells	[68]

*Ac:* acetylation

In contrast, histone acetylation at several endogenous inhibitory proteins, including WIF-1, SFRP, and DKK1, can regulate the expression of these molecules to inhibit Wnt/β-catenin signaling. For example, WIF-1 expression can be silenced by suppression of histone acetylation. Following treatment with trichostatin A (TSA), the expression of WIF1 is restored. Furthermore, HDAC3 may contribute to the restriction of histone acetylation at the promoter of WIF-1 to block its expression [63]. In HCC with hepatitis C virus (HCV) infection, based on HDAC1, the viral core protein could silence SFRP1 expression by inhibiting histone H3 acetylation [64]. After treatment of glioblastoma cells with TSA, the acetylated histone H3 is increased at the promoters of WIF-1, SFRP1, and DKK1 [65]. However, SIRT1 can suppress H3K9 and H4K16 acetylation to restrict SFRP1 and SFRP2 mRNA expression [38].

In breast cancer, it was found that prostate tumor overexpressed-1 (PTOV1) can suppress transcription of DKK1 by recruiting HDAC1 and HDAC2 and decreasing histone H3/H4 acetylation levels at the DKK1 promoter [66]. Li et al. observed that, through initiating acetylation of H3K9 and H3K14 at the DKK1 promoter, GCN5 could promote DKK1 expression to modulate periodontal ligament stem cell differentiation [67]. Furthermore, in breast cancer, chromobox protein homolog 7 (CBX7) is observed to enhance the expression of DKK1. Notably, CBX7 can recruit p300/CBP to the DKK1 promoter to increase histone H3 acetylation [68]. In colon cancer SW480 cells, Genistein was found to affect histone H3 acetylation at the DKK1 promoter [69]. Additionally, Niu et al. observed that epidermal growth factor (EGF) initiates DKK1 expression in HCC cells by increasing histone H3 acetylation through p300 [70] (Table 1). Together, these studies suggest that histone acetylation at the promoters of endogenous inhibitory molecules can promote expression of these gene to suppress Wnt/β-catenin signaling, which means that the deacetylation of histone at the promoters of these molecules is in favor of Wnt/β-catenin signaling.

### Targeting protein acetylation to suppress Wnt/β-catenin signaling

Given that KATs and KDACs can modulate protein acetylation, compounds with the function of blocking the activity of KATs or KDACs have important therapeutic potential for diseases involving dysfunction of protein acetylation. To date, numerous KAT inhibitors and KDAC inhibitors have been discovered, and some of these inhibitors have been approved for tumor treatment or undergone clinical trials to explore their exact clinical effect on a variety of cancers [10, 71]. Our review presented here indicates that not only acetylation but also deacetylation of certain proteins can modulate Wnt/β-catenin signaling. Especially, it has been demonstrated that acetylation or deacetylation of the target molecules is regulated by different KATs or KDACs. It is reasonable to speculate that the use of both KAT inhibitors and KDAC inhibitors can restrict this signaling to facilitate the treatment of various cancers.

Consistent with the above speculation, current data indicate that some KAT inhibitors can suppress this signaling (Table 2). Especially, curcumin [72], the active compound of turmeric or *Curcuma longa* L, with the function of targeting P300, could decrease the growth of HCC cells through regulating the Wnt/β-catenin pathway. Moreover, the clinical applications of curcumin in suppressing multiple cancers, including breast cancer, colorectal cancer, and pancreatic cancer have undergone Phase I/II clinical trials [73]. Garcinol, an extract of the traditional Chinese medicine *Garcinia indicia* [74, 75], also targets P300 and restricts the signaling in non-small cell lung carcinomas and breast cancer cells. However, the therapeutic potential of garcinol in inhibiting different cancers in clinical has not been reported. ICG-001, or the structural derivative PRI-724, can suppress CBP/β-catenin in several tumor cells as well (Table 2). In addition, application of this compound in inhibiting cancer was investigated in a Phase I/II clinical trial [76–86].

**Table 2:** Information on KAT inhibitors and KDAC inhibitors to suppress Wnt/β-catenin signaling

Drug name	KAT or HDAC specificity	Clinical stage in treating cancer	Target cancer cell models	Effect on Wnt/β-catenin signaling	References
Curcumin	KAT inhibitor	Phase I/II	HCC, Breast cancer, Chronic myeloid leukemia, Colorectal cancer, Colon carcinoma, Intestinal adenoma, Pancreatic cancer, Ovarian carcinoma, Head and neck squamous cell carcinoma	Inhibition	[72, 73]
Garcinol	KAT inhibitor	Preclinical	Non-small cell lung carcinomas, Breast cancer	Inhibition	[74, 75]
ICG-001	KAT inhibitor	Phase I/II	Osteosarcoma, Pancreatic cancer, HCC, Nasopharyngeal carcinoma, Uveal melanoma, Colorectal cancer, Lung cancer, glioma, Myeloma, Gastric cancer, Acute lymphoblastic leukemia	Inhibition	[4,76–86]
TSA	HDAC inhibitor	Preclinical	Pituitary adenoma, Colorectal carcinoma, HCC	Inhibition	[10, 87–89]
Sodium butyrate	HDAC inhibitor	FDA approved	Gastric cancers, Colon carcinoma	Inhibition	[10, 90, 91]
Valproic acid	HDAC inhibitor	FDA approved	Glioma, Bladder cancer, Acute T lymphoblastic leukemia	Inhibition	[10, 92–94]
MGCD0103	HDAC inhibitor	Phase II trial	Colon cancer	Inhibition	[10, 95]
Chidamide	HDAC inhibitor	Approved in China	B cell acute lymphocytic leukemia	Inhibition	[10, 96]

Furthermore, several histone deacetylase inhibitors, including TSA [10, 87–89], an inhibitor of class I, II, and IV HDACs, can target Wnt/β-catenin signaling. However, the clinical potential of this compound for the inhibition of cancers is unknown. Sodium butyrate [10, 90, 91] and valproic acid [10, 92–94], both of which target class I, and II HDACs, have been approved by the Food and Drug Administration (FDA), and also can suppress cancer by targeting this signaling. MGCD0103, also known as mocetinostat [95], is an inhibitor of Class I and IV HDACs and underwent a Phase II trial for treating cancers; it has the role of suppressing Wnt/β-catenin signaling as well. Additionally, chidamide [10, 96], an inhibitor of class I and IV HDACs, is approved in China to treat cancer and can target Wnt/β-catenin signaling in cancer (Table 2). However, the molecular mechanisms related to this signaling inhibition mediated by these inhibitors in most cancers are not well clarified.

## Conclusions

The canonical Wnt/β-catenin pathway is a conserved signaling mechanism that modulates a variety of physiological and pathological processes. Especially, canonical Wnt/β-catenin signaling is often hyperactivated in cancers and has a significant role in the occurrence and progression of the disease [1, 2, 97, 98]. The clinical implications of potent drugs targeting this signaling to inhibit different tumors have been assessed [99–101], and the current pharmacological intervention mainly focuses on inhibiting Wnt molecules and their receptors, stabilizing the “destruction protein complex” of β-catenin, blocking the activity of β-catenin, as well as suppressing the interaction of β-catenin with its co-factors [100, 101]. However, the molecular mechanism related to the modulation of Wnt/β-catenin signaling in different types of tumor is complicated. To better target this signaling in clinical treatment, a more thoroughly understanding of the cellular factors that benefit the regulation of this signaling is needed.

As mentioned, protein lysine acetylation involves histone acetylation and non-histone acetylation. Histone acetylation is a vital epigenetic process that critically facilitates the control of gene expression. Additionally, as a very important type of PTM, non-histone acetylation can influence the expression and activity of target proteins. The reviewed studies presented here reveal that Wnt/β-catenin signaling has a significant role in the modulation of two types of protein lysine acetylation. Conversely, protein lysine acetylation not only modulates the expression of important molecules in this signaling by histone acetylation but also directly regulates the function of these signaling-related core proteins to control its activation. To our knowledge, although the available data have demonstrated the cross-regulation between this signaling and protein lysine acetylation as mentioned above, our information on the detailed interaction between the constituents of Wnt/β-catenin signaling with protein lysine acetylation, including histone acetylation as well as non-histone protein acetylation, remains limited. Therefore, additional research focusing on the interplay of Wnt/β-catenin signaling with protein lysine acetylation is required to deeply understand their coordinated roles and related mechanisms.

Generally, protein lysine acetylation relies on the balance of catalytic activity in KATs and KDACs. So far, a variety of KAT inhibitors and KDAC inhibitors have been identified [10, 71], and some of these identified inhibitors have been approved for targeting different cancers in clinical. Moreover, our reviewed studies suggest that many of these inhibitors can inhibit the development of different cancers by blocking this signaling. However, the information on the suppression of this signaling mediated by these inhibitors is mainly from in vitro cell models [71–96]. Data from animal experiments, as well as clinical trials, are needed to confirm whether the anticancer effect of these inhibitors is related to Wnt/β-catenin signaling. Furthermore, given the critical role of lysine acetylation on the modulation of Wnt/β-catenin signaling, a better understanding of the roles and associated mechanisms linked to protein lysine acetylation to facilitate this signaling activation may give us a unique opportunity to treat cancers.

## Data Availability

Not applicable.

## Abbreviations

APC: Adenomatous polyposis coli
BMSCs: Bone marrow-derived mesenchymal stem cells
CK1: Casein kinase 1
KATs: Lysine acetyltransferases
HATs: Histone acetyltransferases
KDACs: Lysine deacetylases
HDACs: Histone deacetylases
HCC: Hepatoma carcinoma
BMSCs: Bone marrow-derived mesenchymal stem cells
HCV: Hepatitis C virus
LEF: Lymphoid enhancer factor
GSK3β: Glycogen synthase kinase 3β
β-TRCP: Beta-transducin repeat-containing protein
FZD: Frizzled proteins
LRP: Lipoprotein receptor-related protein
TCF: T cell factor
WIF-1: Wnt inhibitory factor 1
DKK: Dickkopf-related protein
SFRPs: Secreted frizzled-related proteins
TAT1: Tubulin N-acetyltransferase 1
CBP: CREB-binding protein
GCN5: General Control Non-repressed 5
TIP60: 60 KDa Tat-interactive protein
ULK1: Unc51-like kinase-1
BOP1: Block of proliferation 1
FOXP1: Forkhead box protein P1
CREPT: Cell-cycle related and expression-elevated protein in tumor
ACLY: ATP citrate lyase
PCAF: P300/CBP-associated factor
KLF4: Kruppel-like factor 4
NFAT5: Nuclear factor of activated T-cells 5
LCSCs: Liver cancer stem cells
Bcl-3: B-cell lymphoma 3
Pygo2: Pygopus 2
AKIP1: A-kinase interacting protein 1
Barx2: ArH-like Homeobox 2
Pax7: Paired box 7
GRIP-1: Glutamate receptor interacting protein 1
MEF: Mouse embryonic fibroblasts
PTOV1: Prostate tumor overexpressed-1
CBX7: Chromobox protein homolog
